# Case Report: Primary Hyperparathyroidism due to Posterior Mediastinal Parathyroid Adenoma With One-Year Follow-Up

**DOI:** 10.3389/fsurg.2022.893259

**Published:** 2022-05-31

**Authors:** Yi Shao, Qingdong Zeng, Bin Lv, Xu Chen, Lei Sheng

**Affiliations:** ^1^ Department of Thyroid Surgery, General Surgery, Qilu Hospital of Shandong University, Jinan, China; ^2^ Department of Pathology, Qilu Hospital of Shandong University, Jinan, China

**Keywords:** ectopic parathyroid adenoma, primary hyperparathyroidism, posterior superior mediastinum, thoracic surgery, vitamin D deficiency

## Abstract

Ectopic parathyroid adenoma, though rare, is one of the causes of persistent hyperparathyroidism and recurrence of hyperparathyroidism. Ectopic parathyroid glands can be seen in thymus, thyroid, and mediastinum. However, ectopic parathyroid adenoma occurred in the posterior superior mediastinum is extremely rare. Here, we report a case of primary hyperparathyroidism caused by ectopic parathyroid adenoma located in the posterior superior mediastinum. Serum parathyroid hormone, calcium, and vitamin D levels of the patient was followed up for one year.

## Introduction

Primary hyperparathyroidism (PHPT) is a disease characterized by an excessive secretion of parathyroid hormone, leading to osteoporosis, ureteral calculi, and serum hypercalcemia. Ninety percent of parathyroid cases were caused by a single parathyroid adenoma, and the less common causes included multiple hyperplasia of the gland (6%), double adenoma (4%), and parathyroid carcinoma (<1%) (1). In about 6–16% of cases, one or more hyperparathyroidism adenoma is located in the ectopic position (2). Mediastinum is less common as an ectopic site, accounting for 1–2% of the cases (3). Among mediastinal ectopic parathyroid gland adenoma, posterior mediastinal ectopic parathyroid gland adenoma was extremely rare in literature reports. Here, we reported a case of PHPT due to posterior mediastinal ectopic parathyroid gland adenoma with one-year follow-up.

## Case Description

### Patient Information

A 46-year-old female patient had a history of elevated alkaline phosphatase (AKP) for 4 years. She had occasional thirst, with no abdominal pain, abdominal distension, acid reflux, heartburn, constipation, dyspepsia, or nocturia. The history of renal insufficiency was denied. The patient had regular physical examination every year and found that his AKP level increased progressively. Osteoporosis was found by physical examination two years ago with no bone pain or fracture history. The patient’s menstruation is still regular, and there is no obvious history of dysmenorrhea.

### Diagnostic Assessment

**Figure 1:**
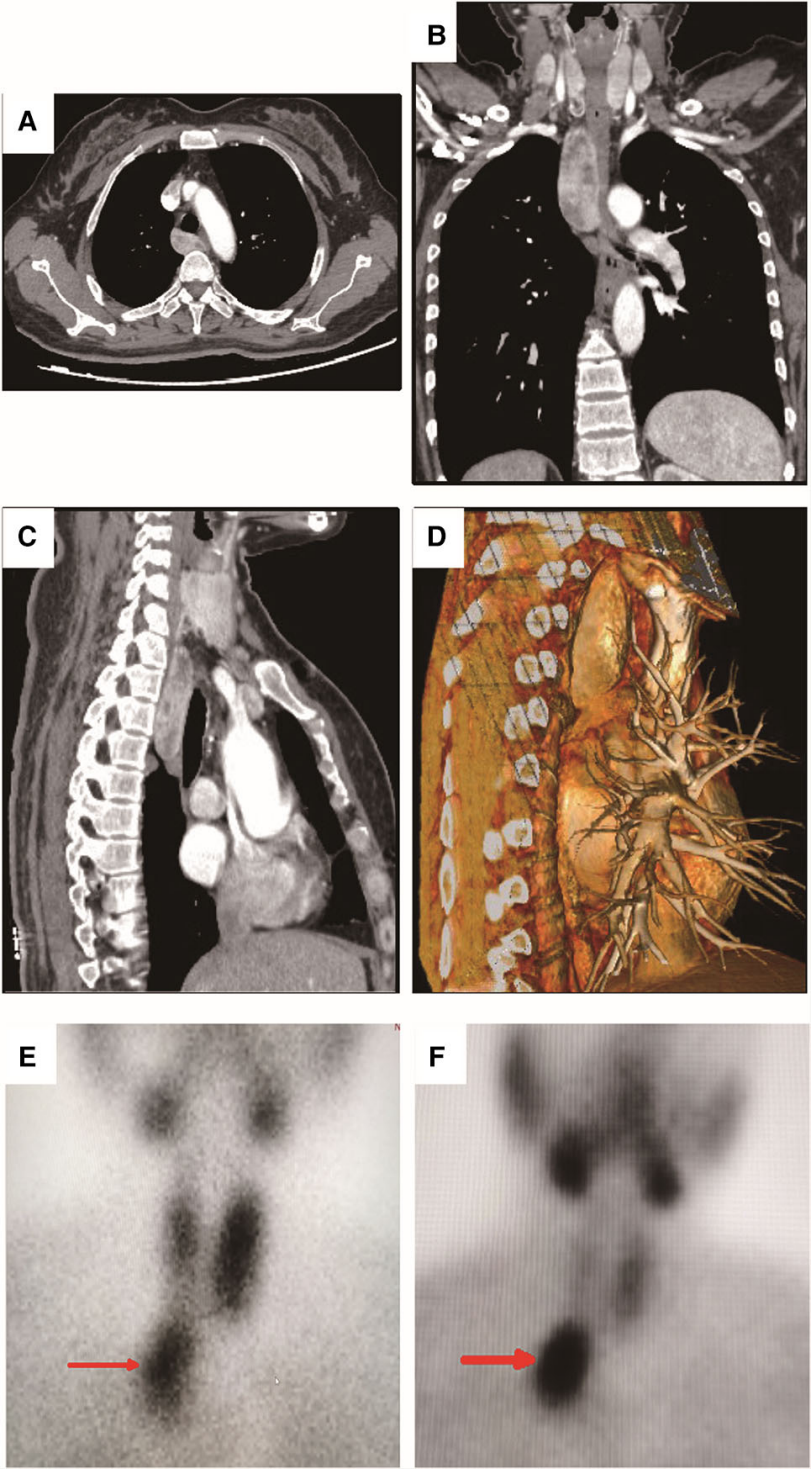
**Computed tomography (CT) scan and 99^m^-Technetium sestamibi scanning of the patient.** (**A**) Transverse view of the CT scanning showed a mixed density mass with uneven enhancement in the posterior mediastinum. The maximum cross-section size was about 3.3×1.9 cm.; (**B**) Coronal view of the CT scanning; (**C**) Sagittal view of the CT scanning; (**D**). Three-dimensional reconstruction in sagittal view of the CT scanning; (**E**) The 15th minute static imaging: the left thyroid lobe was more prominent, whereas the uptake of imaging agent in the right thyroid lobe was decreased; In addition, localized abnormal uptake of imaging agent under the right thyroid lobe was found (as shown by the red arrow). (**F**) The two-hour delayed imaging: the uptake of thyroid imaging was lower than that before, the abnormal imaging agent concentration under the right thyroid lobe was clearly displayed (as shown by the red arrow).

Blood test results one month before operation showed that AKP was 294 U/L (normal range 30–100 U/L), PTH 981.20 pg/mL (normal range 15–65 pg/mL), calcium 2.93 mmol/L (normal range 2.00–2.60 mmol/L), phosphorus 0.64 mmol/L, Vitamin D 6.97 ng/mL (normal range ≥30 ng/mL). Right forearm bone mineral density detection showed BMD 0.391, Z score −5.33, and t score −5.44. Computed tomography scan showed posterior superior mediastinal was occupied with a cystic solid tumor whose maximum cross-section size was about 3.3 × 1.9 cm (Figure 1A–1D). No special signs were found by neck ultrasound. The patient underwent 99mTechnetium sestamibi scanning, indicating a mass with increased uptake of imaging agent in the posterior mediastinum (Figure 1E–1F).

### Therapeutic Intervention

**Figure 2:**
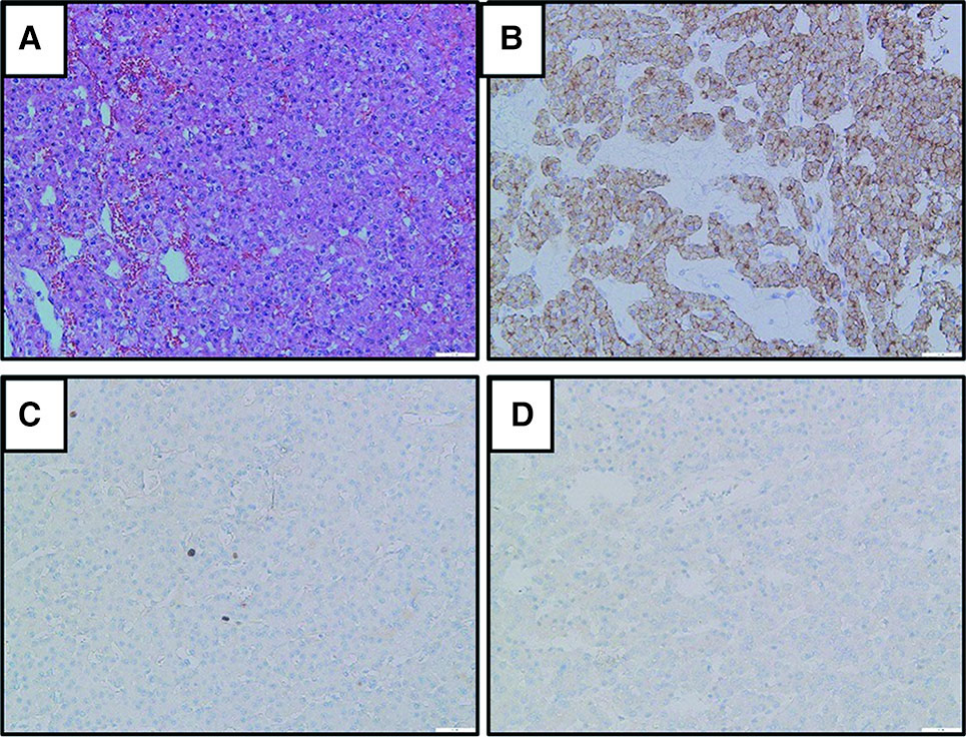
**Histology and immunohistochemistry of parathyroid adenoma.** (**A**). Hematoxylin and eosin staining. (**B**) Positivity for parathyroid hormone. (**C**) About 2% of positivity for Ki-67 staining. (**D**) Negativity for thyroid transcription factor 1 (TTF-1). Magnification ×200.

The patient had daily subcutaneous injection of a total amount of 100–300 IU per day of salmon calcitonin preoperatively due to hypercalcemia and underwent single-port thoracoscopic resection of the mediastinal tumor. The tumor was carefully removed by ultrasonic scalpel. The volume of the tumor was about 6.0 × 4.2 × 1.0 cm at pathological examination. Postoperative pathology confirmed parathyroid adenoma (Figure 2).

## Follow-up and Outcomes

**Figure 3:**
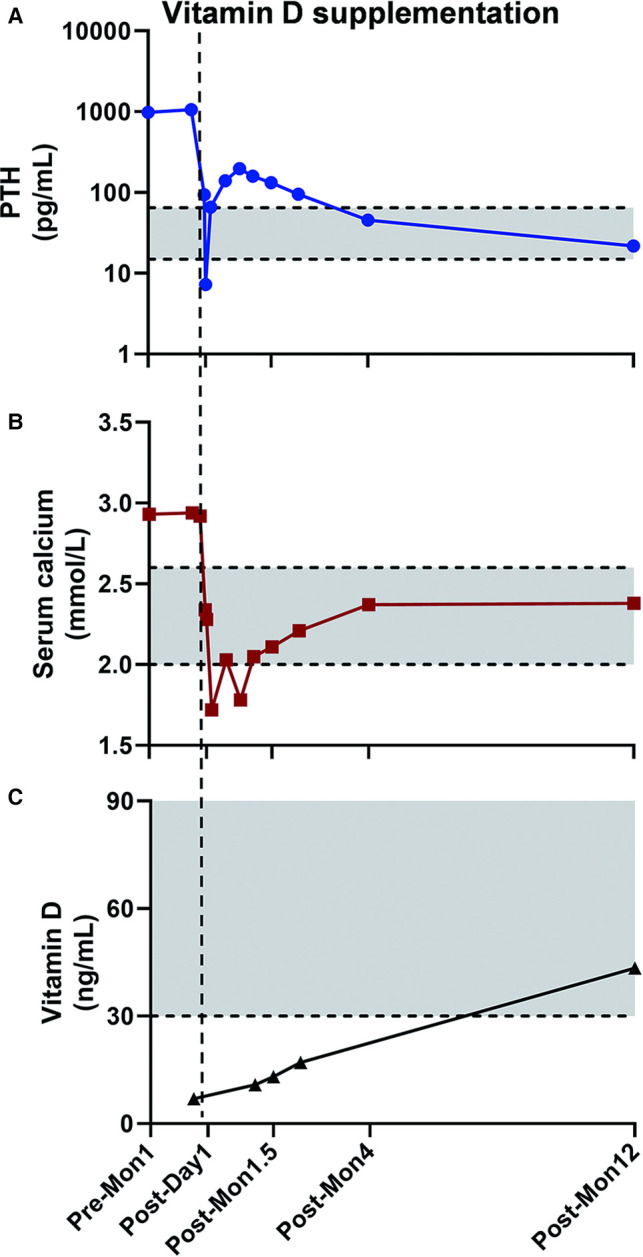
**The PTH, serum calcium, and Vitamin D level changed perioperatively.** Grey area was the normal range PTH (15–65 pg/mL), serum calcium (2.00–2.60 mmol/L), and Vitamin D level (≥30 ng/mL) defined at our center. The PTH level decreased immediately after the operation but went up at the early follow-up (**A**), while the postoperative blood calcium level was lower than the upper limit of normal value (**B**). After vitamin D levels were normalized with vitamin D supplementation (**C**), the PTH level went back to normal level at the one year of follow-up. Pre-Mon 1: one month prior to surgery. Post-Day 1: one day after surgery. Post-Mon 1.5, Post-Mon 4, and Post-Mon 12: 1.5, 4, and 12 months after surgery.

The PTH and blood calcium levels decreased instantly after the operation. The PTH level on the first day after the operation was 93.96 pg/mL, and on the second day after the operation was 7.29 pg/mL. However, on the fourth day after the operation, the PTH level raised to 65.61 pg/mL (normal range 15–65 pg/mL) and continued to rise to 159.2 pg/mL one month after the operation. Serum calcium concentration was still within the normal level and no specific symptoms were shown. Meanwhile, the vitamin D level was found lower than the normal level in the first month after surgery. So the patient was supplemented with vitamin D 800 IU per day. The PTH level continues to decrease, whereas vitamin D levels continue to rise ever since exogenous vitamin D was supplemented. One year after the operation, serum PTH, calcium, and vitamin D levels returned to normal (Figure 3).

### Timeline

**Figure 4:**
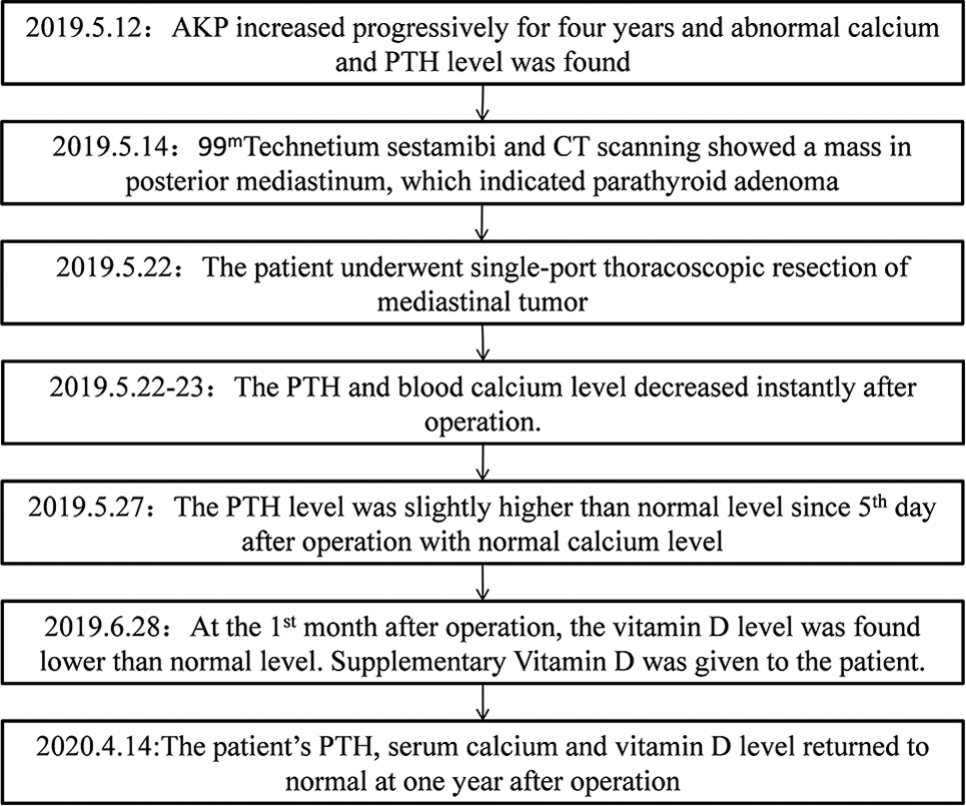
**The timeline of clinical interventions of this patient**.

The timeline of clinical interventions for this patient was shown in Figure 4.

## Discussion

PHPT is a very common endocrine disease characterized by inappropriate secretion of parathyroid hormone in the absence of external stimulation. 80%–95% of patients with PHPT can be cured by parathyroidectomy after the first operation (4). Patients who were not cured had hypercalcemia immediately after operation, or hypercalcemia after long-term normal blood calcium level. The failure of parathyroidectomy is mainly due to inadequate resection of parathyroid adenoma or the presence of a second adenoma, parathyroid hyperplasia (usually with familial PHPT syndrome), or parathyroid carcinoma. A common and main reason for persistent PHPT is the ectopic location of the parathyroid, which is the result of abnormal migration of the parathyroid in early fetal development (5).

The parathyroid glands originate from the endoderm and develop from the dorsal wing of the third and fourth pharyngeal pouch (6). Embryologically, the upper parathyroid originates from the fourth-gill sac and migrates caudally with the thyroid gland, while the lower parathyroid originates from the third-gill sac and migrates with the thymus gland. The distribution area of the superior parathyroid gland is narrow, and it is stably distributed in the fat tissue around the thyroid gland, near the route of the recurrent laryngeal nerve until it enters the cricothyroid muscle. On the contrary, the inferior parathyroid gland is distributed in a wide area of thyrothoracic ligament and pretracheal fat tissue, around the lower pole of the thyroid gland (6, 7). Autopsy confirms that the incidence of ectopic parathyroid is between 2% and 42.8% (8, 9). Ectopic parathyroid adenomas may be located anywhere from the base of the tongue to the mediastinum. The most common location of ectopic lower parathyroid is in the anterior mediastinum, which is related to thymus or thyroid, while the most common location of ectopic upper parathyroid is in tracheoesophageal sulcus and posterior esophagus (10, 11). Posterior superior mediastinum is a rare ectopic location of the superior parathyroid gland (10), which was rarely reported in the literature.

In suspected cases of ectopic parathyroid adenoma, neck ultrasound (US) and 99mTechnetium sestamibi scanning (MIBI) are the preferred imaging methods. The US can effectively detect the adenoma around the lower pole of the thyroid, but it is not effective to show the ectopic parathyroid adenoma in the posterior or upper mediastinum (12). Ectopic parathyroid can be detected by MIBI with the sensitivity almost the same as that of *in situ* parathyroid adenoma (13). In most studies, the sensitivity of ultrasound alone as a single method to identify ectopic adenoma was 27–89%, while MIBI alone was 80–90% (14–16).

For patients with posterior superior mediastinal ectopic parathyroid adenoma, video-assisted thoracoscopy (VATS) combined with intraoperative MIBI scan and parathyroid hormone assessment can minimize the surgical anatomy and avoid median sternotomy and thoracotomy. It has been suggested that parathyroid hormone levels should be assessed within 15 min after parathyroidectomy, and should be reduced to at least 50% of that before operation. If PTH continues to be higher than normal, ectopic or multiglandular disease is suspected (17). However, the definition of curative parathyroidectomy is: blood calcium is normal 6 months after the operation, and the definition of recurrence of hyperparathyroidism is: hypercalcemia caused by hyperparathyroidism occurs again 6 months after blood calcium is normal (18). Wang found that in 50% of the patients whose parathyroid adenoma had been successfully removed, blood calcium decreased by 2–3 mg in the first 24 h after surgery. Unless bone starvation syndrome occurs, the initial decrease in blood calcium can be restored 3–4 days after surgery and the blood calcium is within the normal range (19). In patients with high serum AKP level and osteoporosis before the operation, bone starvation syndrome and lower-than-expected blood calcium level may appear after the operation. This is consistent with the patient’s condition. Under normal circumstances, parathyroid hormone should be decreased after successful parathyroid surgery. However, abnormally high PTH level with normal calcium level after the operation can be seen in certain percentage of patients whose parathyroid adenoma have successfully been removed, accounting for 8%–43% of patients (20–22). 78%–88% of the patients with elevated postoperative PTH levels can eventually decrease to normal range or lower within 12–16 months. However, some reports showed that PTH level could still be elevated, with normal blood calcium level, 1–4 years after operation (23, 24).

Abnormally high postoperative PTH level is more common in the following cases: ① Severe preoperative hyperparathyroidism (such as higher PTH level, larger glands, and multiple gland diseases); ② Decreased sensitivity of peripheral tissue to PTH (possibly due to chronic long-term increase of PTH before operation); ③ Renal insufficiency; ④ Exaggerated reflection of excessive decalcification of bone after operation; and ⑤Preoperative vitamin D deficiency. Vitamin D deficiency is more common in elderly patients and female patients (20, 23, 24). Therefore, for most patients, higher postoperative PTH does not mean the failure of surgery, nor is it a good indicator of postoperative disease status. Charlett reported that the sensitivity and specificity of abnormal serum PTH level to predict surgical failure were 62.1% and 75%, respectively (25). Some researchers suggested that detection of PTH level in patients with PHPT after surgery may mislead the treatment. For patients with normal blood calcium, PTH monitoring is not essential (26). Others recommended that the blood calcium level should be reexamined regularly after the operation, but not PTH. If PTH is detected and higher than normal level, then vitamin D level should also be detected in case of existence of vitamin D deficiency (25, 26).

In this case, vitamin D deficiency may be the cause of elevated PTH levels. Vitamin D in circulation binds with vitamin D binding protein, transports to liver, and generates 25 hydroxyvitamin [25–(OH) D_3_] under the action of 25 hydroxylase. 25–(OH) D_3_ is the main form of vitamin D in human blood circulation with no biological activity, and it is transformed into 1, 25–(OH) _2_D_3_, the active form of vitamin D, under the action of 1α-hydroxylase in kidney. 1, 25–(OH) _2_D_3_ can reduce PTH synthesis and secretion through negative feedback. On the contrary, the parathyroid hormone can increase the renal tubular reabsorption of calcium and promote the renal production of 1, 25–(OH) _2_D_3_, and promote the conversion of 25–(OH) D_3_ to 1, 25–(OH) _2_D_3_ (27). In this case, the vitamin D deficiency before operation and the failure of early exogenous supplementation may be the main reason for the sustained elevation of PTH level after the operation. After vitamin D was supplemented, the PTH level of this patient gradually returned to normal, which also verified our conjecture. In addition, we speculated that the long history of abnormally high PTH level may cause decreased sensitivity to PTH in peripheral tissue, which is plausibly a contributing factor for increased postoperative PTH level.

## Data Availability

The original data presented in the study are included in the article, further inquiries can be directed to the corresponding author.

## References

[1] Ruda JM Hollenbeak CS Stack BC Jr. A systematic review of the diagnosis and treatment of primary hyperparathyroidism from 1995 to 2003. Otolaryngol Head Neck Surg. (2005) 132(3):359–72. 10.1016/j.otohns.2004.10.005 15746845

[2] Roy M Mazeh H Chen H Sippel RS. Incidence and localization of ectopic parathyroid adenomas in previously unexplored patients. World J Surg. (2013) 37(1):102–6. 10.1007/s00268-012-1773-z 22968537

[3] Amer K Khan AZ Rew D Lagattolla N Singh N. Video assisted thoracoscopic excision of mediastinal ectopic parathyroid adenomas: a UK regional experience. Ann Cardiothorac Surg. (2015) 4(6):527–34. 10.3978/j.issn.2225-319X.2015.09.04 26693148 PMC4669253

[4] Marcocci C Cetani F. Clinical practice: primary hyperparathyroidism. N Engl J Med. (2011) 365(25):2389–97. 10.1056/NEJMcp1106636 22187986

[5] Kelly HR Hamberg LM Hunter GJ. 4D-CT for preoperative localization of abnormal parathyroid glands in patients with hyperparathyroidism: accuracy and ability to stratify patients by unilateral versus bilateral disease in surgery-naive and re-exploration patients. AJNR Am J Neuroradiol. (2014) 35(1):176–81. 10.3174/ajnr.A3615 23868155 PMC7966477

[6] Mohebati A Shaha AR. Anatomy of thyroid and parathyroid glands and neurovascular relations. Clin Anat. (2012) 25(1):19–31. 10.1002/ca.21220 21800365

[7] Fancy T Gallagher D 3rd Hornig JD. Surgical anatomy of the thyroid and parathyroid glands. Otolaryngol Clin North Am. (2010) 43(2):221–7. 10.1016/j.otc.2010.01.001 20510710

[8] Wang C. The anatomic basis of parathyroid surgery. Ann Surg. (1976) 183(3):271–5. 10.1097/00000658-197603000-00010 1259483 PMC1344236

[9] Hojaij F Vanderlei F Plopper C Rodrigues CJ Jacomo A Cernea C Parathyroid gland anatomical distribution and relation to anthropometric and demographic parameters: a cadaveric study. Anat Sci Int. (2011) 86(4):204–12. 10.1007/s12565-011-0111-0 21850415

[10] Phitayakorn R McHenry CR. Incidence and location of ectopic abnormal parathyroid glands. Am J Surg. (2006) 191(3):418–23. 10.1016/j.amjsurg.2005.10.049 16490559

[11] Mendoza V Ramirez C Espinoza AE Gonzalez GA Pena JF Ramirez ME Characteristics of ectopic parathyroid glands in 145 cases of primary hyperparathyroidism. Endocr Pract. (2010) 16(6):977–81. 10.4158/EP10052 20497936

[12] Beierwaltes WH. Endocrine imaging: parathyroid, adrenal cortex and medulla, and other endocrine tumors. Part II. J Nucl Med. (1991) 32(8):1627–39. 1869992

[13] Castellani M Reschini E Longari V Paracchi A Corbetta S Marotta G Role of Tc-99 m sestamibi scintigraphy in the diagnosis and surgical decision-making process in primary hyperparathyroid disease. Clin Nucl Med. (2001) 26(2):139–44. 10.1097/00003072-200102000-00010 11201472

[14] McHenry CR. What’s new in general surgery: endocrine surgery. J Am Coll Surg. (2002) 195(3):364–71. 10.1016/s1072-7515(02)01307-8 12229945

[15] Johnston LB Carroll MJ Britton KE Lowe DG Shand W Besser GM The accuracy of parathyroid gland localization in primary hyperparathyroidism using sestamibi radionuclide imaging. J Clin Endocrinol Metab. (1996) 81(1):346–52. 10.1210/jcem.81.1.8550776 8550776

[16] Friedman K Somervell H Patel P Melton GB Garrett-Mayer E Dackiw AP Effect of calcium channel blockers on the sensitivity of preoperative 99mTc-MIBI SPECT for hyperparathyroidism. Surgery. (2004) 136(6):1199–204. 10.1016/j.surg.2004.06.047 15657576

[17] Phillips IJ Kurzawinski TR Honour JW. Potential pitfalls in intraoperative parathyroid hormone measurements during parathyroid surgery. Ann Clin Biochem. (2005) 42(Pt 6):453–8. 10.1258/000456305774538283 16259796

[18] Wilhelm SM Wang TS Ruan DT Lee JA Asa SL Duh QY The American association of endocrine surgeons guidelines for definitive management of primary hyperparathyroidism. JAMA Surg. (2016) 151(10):959–68. 10.1001/jamasurg.2016.2310 27532368

[19] Wang CA. Surgical management of primary hyperparathyroidism. Curr Probl Surg. (1985) 22(11):1–50. 10.1016/0011-3840(85)90019-x 4085260

[20] Yamashita H Noguchi S Moriyama T Takamatsu Y Sadanaga K Uchino S Reelevation of parathyroid hormone level after parathyroidectomy in patients with primary hyperparathyroidism: importance of decreased renal parathyroid hormone sensitivity. Surgery. (2005) 137(4):419–25. 10.1016/j.surg.2004.12.009 15800489

[21] Mizrachi A Gilat H Bachar G Feinmesser R Shpitzer T. Elevated parathyroid hormone levels after parathyroidectomy for primary hyperparathyroidism. Head Neck. (2009) 31(11):1456–60. 10.1002/hed.21119 19405085

[22] Carneiro DM Solorzano CC Irvin GL 3rd., Recurrent disease after limited parathyroidectomy for sporadic primary hyperparathyroidism. J Am Coll Surg. (2004) 199(6):849–53. 10.1016/j.jamcollsurg.2004.08.013 15555964

[23] Solorzano CC Mendez W Lew JI Rodgers SE Montano R Carneiro-Pla DM Long-term outcome of patients with elevated parathyroid hormone levels after successful parathyroidectomy for sporadic primary hyperparathyroidism. Arch Surg. (2008) 143(7):659–63. 10.1001/archsurg.143.7.659 18645108

[24] Nordenstrom E Westerdahl J Isaksson A Lindblom P Bergenfelz A. Patients with elevated serum parathyroid hormone levels after parathyroidectomy: showing signs of decreased peripheral parathyroid hormone sensitivity. World J Surg. (2003) 27(2):212–5. 10.1007/s00268-002-6600-5 12616439

[25] Charlett SD Aye M Atkin SL England RJ. Defining failure after parathyroidectomy for primary hyperparathyroidism: case series. J Laryngol Otol. (2011) 125(4):394–8. 10.1017/S0022215110002562 21208475

[26] Yen TW Wilson SD Krzywda EA Sugg SL. The role of parathyroid hormone measurements after surgery for primary hyperparathyroidism. Surgery. (2006) 140(4):665–72. 10.1016/j.surg.2006.07.006 17011915

[27] Holick MF. Vitamin D deficiency. N Engl J Med. (2007) 357(3):266–81. 10.1056/NEJMra070553 17634462

